# Temporal properties of higher-order interactions in social networks

**DOI:** 10.1038/s41598-021-86469-8

**Published:** 2021-03-29

**Authors:** Giulia Cencetti, Federico Battiston, Bruno Lepri, Márton Karsai

**Affiliations:** 1 Mobs Lab, Fondazione Bruno Kessler, Via Sommarive 18, 38123 Trento, Italy; 2 Department of Network and Data Science, Central European University, 1100 Vienna, Austria

**Keywords:** Computational science, Scientific data, Complex networks, Nonlinear phenomena

## Abstract

Human social interactions in local settings can be experimentally detected by recording the physical proximity and orientation of people. Such interactions, approximating face-to-face communications, can be effectively represented as time varying social networks with links being unceasingly created and destroyed over time. Traditional analyses of temporal networks have addressed mostly pairwise interactions, where links describe dyadic connections among individuals. However, many network dynamics are hardly ascribable to pairwise settings but often comprise larger groups, which are better described by higher-order interactions. Here we investigate the higher-order organizations of temporal social networks by analyzing five publicly available datasets collected in different social settings. We find that higher-order interactions are ubiquitous and, similarly to their pairwise counterparts, characterized by heterogeneous dynamics, with bursty trains of rapidly recurring higher-order events separated by long periods of inactivity. We investigate the evolution and formation of groups by looking at the transition rates between different higher-order structures. We find that in more spontaneous social settings, group are characterized by slower formation and disaggregation, while in work settings these phenomena are more abrupt, possibly reflecting pre-organized social dynamics. Finally, we observe temporal reinforcement suggesting that the longer a group stays together the higher the probability that the same interaction pattern persist in the future. Our findings suggest the importance of considering the higher-order structure of social interactions when investigating human temporal dynamics.

## Introduction

Complex networks are fundamental tools to represent complex systems made of interacting units, with applications in biology, social sciences, transport infrastructures, communications, financial markets, and more^1–4^. Incorporating a set of discrete nodes and the connections between them, the networks schematize the existing relationships among agents, providing a synthetic picture of the system architecture. Despite the success of network representations of complex systems in the last thirty years, static graphs fall short to effectively describe a wide variety of real world systems, especially when the dynamics of their structural changes are in focus. In networked systems, whether nodes represent people, cells, neurons, virtual or physical sites, their interactions are not bounded to be static but are rather evolving, with nodes and links, which appear and disappear over time. To address the time-varying aspect of complex structures, the field of temporal networks emerged^5–8^ providing useful representations and tools to study the dynamics of real complex systems. The framework is particularly suited to describe social systems where coupling contacts among people naturally change over time in online and offline social networks, email and mobile phone communications, and more^9–13^. However, social interactions may vary over multiple temporal scales, ranging from long lasting friendships to accidental interactions between strangers. Moreover, consecutive interactions may not appear independently but follow each other rapidly forming bursty patterns^14^ potentially due to intrinsic correlations^15^ or simply via circadian fluctuations of human activity^16^. Temporal networks describe such processes at the highest time resolution to understand how single interactions may lead to collective phenomena, as long trains of bursty events, or the emergence of the complex social structure.

Network and statistical physics approaches were originally devised to describe dyadic relationships^17,18^ and can only provide a limited representation of systems interacting beyond pairwise schemes. Such higher-order interactions are ubiquitous^19^, from human to technological and biological systems^20,21^. For instance scientific authors naturally team up in larger groups to complement the expertise of different members^22^, neurons send and receive stimuli from multiple adjacent partners at the same time^21,23^, and the stability of large ecosystems relies on mutual and cooperative partnerships often involving three or more species^24,25^. Besides, higher-order interactions were shown to significantly modify the collective behavior of many dynamical processes, from diffusion^26,27^ and synchronization^28–30^ to spreading^31,32^, social dynamics^33,34^ and games^35^. For a thorough introduction on the structure and dynamics of these higher-order systems, we refer the interested reader to the comprehensive overview provided in Ref^19^.

In this paper our goal is to study the heterogeneous dynamics of group interactions by looking at bursty patterns of higher-order structures in temporal networks. We analyze the temporal properties of multi-party face-to-face interactions^36^ recorded in the *Sociopatterns * project^37^, and co-location contacts recorded by Bluetooth technology in the Copenhagen Network Study (*CNS*)^38^ and in the Friends and family (*F&F*) project^39^. We define group interactions in this setting and determine the number of groups to classify them according to their size. By analyzing their duration and the time between their subsequent appearances we identify long bursty trains of recurrent group interactions due to temporal correlations.

Finally, we investigate the temporal evolution of groups and how their size changes over time by progressively acquiring or losing members, observing a reinforcement of group structures over time. Our results generalise universal phenomena earlier observed for dyadic interactions^40^ for the case of higher-order temporal structures.

## Results

### Temporal higher-order social interactions

We aim at investigating the temporal dynamics of the higher-order structure of human proximity interactions in different social settings. To this end, we choose five datasets: three from the *Sociopatterns * project, which describe face-to-face interactions^36,37^ (a) in an office building in France^41^ over 11 days; (b) in a hospital ward between patients, medical doctors, nurses and administrative staff over 72 h^42^; and (c) during 32 h in a scientific conference^37^. All these three datasets contain dyadic face-to-face interactions with time resolution of 20 s. Analogously, with a lower time resolution (300 s), the fourth dataset, *CNS*, describes the co-location activity of 706 students at the campus of the Technical University of Denmark for one month, recorded by the exchange of Bluetooth radio packets between smartphones. Finally, the fifth dataset that we consider, *F&F*, has been recorded in a young-family residential living community adjacent to a major research university in North America. All members of the community are couples, and at least one of the members is affiliated with the university, for a total of 130 adults for 15 months.

There is a major difference between the *Sociopatterns * datasets and the other two. While the former ones have been collected via wearable devices using RFID technology, the other two datasets, *CNS* and *F&F*, use Bluetooth proximity technology. Thus, in one way, the *Sociopatterns * data record face-to-face contacts, that we can consider as a good proxy for actual interactions between users. On the other way, the Bluetooth technology used for *CNS* and *F&F* records co-location data, registering an interaction event every time that two people are close enough that their devices can detect a signal. Hence, the records of co-locations may provide a weaker proxy of real direct social interactions. The different methods of collecting data result in slightly different dynamics of interactions, as we will analyze in the following. Moreover, since the *CNS* dataset contains metadata like the Bluetooth signal strength for each interaction, which decreases at increasing the distance between the two users, it is possible to cut all the contacts which appear with a signal strength below a certain threshold, in order to make this dataset more comparable with face-to-face interactions (which only capture very close contacts). The threshold that we chose is − 80 rssi, roughly corresponding to 1.5–2 m distance (the same distance reached by the radio signals of *Sociopatterns*^41^). In our coming analysis we concentrate on this pruned dataset, while reporting the corresponding statistics on the complete dataset in the Supplementary Information. Each dataset records the dyadic contacts of people with a specific time resolution $t_{res}$, but it also identifies simultaneous contacts of the participants thus allowing for the observation of group interactions.

Originally exploited for pairwise network analysis, the fine grained temporal structure of these interactions allows us to reconstruct the formation, presence, and deletion of higher-order groups. In the traditional network formalism, a dyadic temporal interaction between two people *a* and *b* at time *t*, which lasted for duration *d*, is represented by a temporal link $e=(a,b,t,d)$. In this setting, the sequence of temporal events builds up a temporal network $G_T=(V_T,E_T,T)$, where any node $a\in V_T$, any event $e\in E_T$ and the network evolve over *T* period, thus $0\le t \le T$ and $0\le d \le T$.

**Figure 1:**
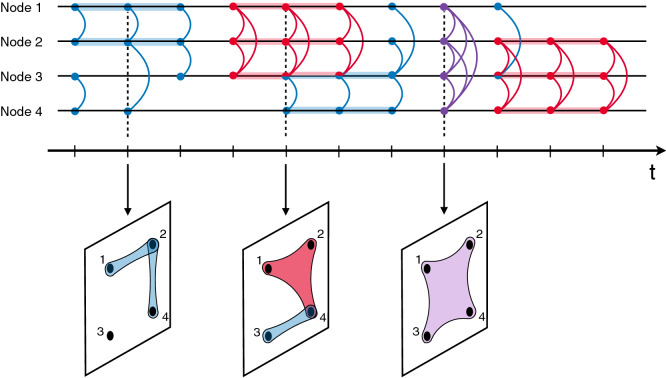
From higher-order interactions to temporal hypergraph. Example of higher-order interactions among a group of four people. The horizontal lines represent the temporal behavior of each individual and curved lines bridge the *n* nodes involved in one interaction, with different colors for different sizes: blue for interaction between two people (2-hyperedges), red for three people (3-hyperedges) and purple for four people (4-hyperedges). Coloured lines on individual timelines indicate the time and duration of interactions, with colors coding their size. Snapshots indicate corresponding hypergraphs at specific times. Note that it is possible to observe open structures, like for instance open triads, i.e. one person interacting with two people that are not connected to each other.

However, people often connect in larger groups, where more than two individuals interact at the same time. Simple links, by definition describing dyadic connections, are not suited to describe such higher-order interactions, which require different types of building blocks, known as *hyperedges*. An *n*-hyperedge, or hyperedge of size *n*, describes an interaction of *n* individuals. In more mathematical terms this is denoted by a simplex of order $n-1$^19^. Simple dyadic links represent the first non-trivial interaction, described by a 2-hyperedge. For temporal data, we define the interaction between a group of *n* people, $i_1, \ldots , i_n$, at time *t* and for duration *d* as a temporal *n*-hyperedge assigned as $e_{n}=(i_1, \ldots , i_n,t,d)$. The sequence of temporal events builds up a temporal hypergraph $H_T=(V_T,E_T,T)$, where any node $i\in V_T$, any event $e_n\in E_T$ (now describing a set of hyperedges) and the hypergraph evolve over *T* period, thus $0\le t \le T$ and $0\le d \le T$. An example of a temporal hypergraph is shown in Fig. 1, where the connections that nodes undertake are coloured according to their size shown with some instantaneous snapshots of the temporal hypergraph underneath.

**Figure 2:**
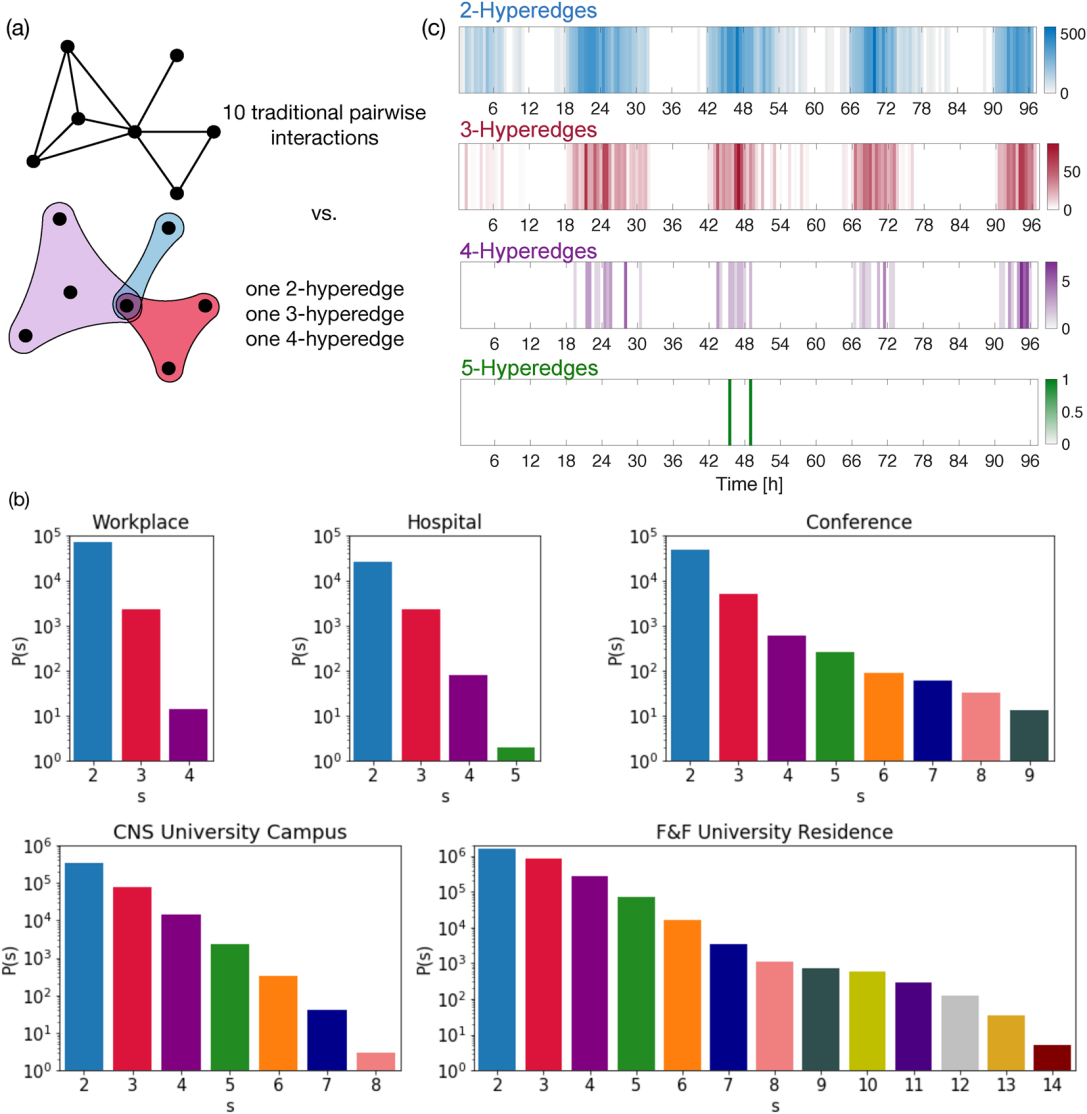
Higher-order structure of temporal human interactions. (**a**) Example of network depicted in a traditional way or as a hypergraph. In the last case interactions of different sizes correspond to different colors: blue for 2-hyperedges (two nodes), red for 3-hyperedges (three nodes), purple for 4-hyperedges (four nodes). (**b**) Histograms reporting the counts of interactions for each different size in the five datasets of human communication: a workplace, an hospital, a scientific conference, and two university campuses. (**c**) Time series of interactions in the hospital dataset represented by hyperedges of size2, 3, 4, and 5.

In the considered datasets each interaction is originally stored through simple links. However, they do not necessarily represent the original building blocks of the interactions. The fine-grained temporal nature of the datasets allows us to reconstruct the original higher-order features of the connections and the corresponding hyperedges. In practice, if at a time *t* there are $n*(n+1)/2$ dyads between the members of a set of *n* nodes such that they form a fully connected clique, we promote the $n*(n+1)/2$ links to a *n*-hyperedge. For instance, if at time *t*, *a* is interacting with *b* and *c*, and *b* is interacting with *c* too, the interactions will be stored into an 3-hyperedge. Note that the same reconstruction is not possible from temporally-aggregated data, where the presence of a closed triangle may be the byproduct of the temporal aggregation of distinct truly pairwise interactions. This is demonstrated in Fig. 2a where the schematic representation of a network is compared with its hypergraph version. The traditional network is characterized by ten simple links, while considering their simultaneous group interactions, we identify one 2-hyperedge (in blue), one 3-hyperedge (in red), and one 4-hyperedge (in purple).

In the following when we refer to a group interaction of size *n*, it corresponds to a maximal clique of size *n*, or in other words an *n*-hyperedge, without being a part of any larger group.

### Statistics of higher-order interactions

The statistics of *maximal* higher-order interactions for the three datasets are reported in Fig. 2b. Smaller interactions involving less people are more numerous in all datasets, however different settings are characterized by different statistics: for instance, among the *Sociopatterns * datasets, the conference reveals the presence of very large aggregations, with up to events of size 9, while in the hospital and workplace settings the group sizes are limited to 5 and 4 respectively. Note also that the workplace dataset was collected for a longer period than the hospital and the conference ones, thus it represents the most connected aggregated network among the *Sociopatterns * datasets and the one with the largest total number of interactions. However, these interactions are mainly pairwise, as shown in Fig. 2, which is particularly peaked at $s=2$, while interactions of size 4 are poorly represented (less than 20 in 11 days). In addition, the workplace network has the highest ratio (nearly one and half orders of magnitude) between the number of dyadic and triplet interactions. This suggests that the workplace network is “the lowest-order”, especially as compared to the conference network, which instead appears to be “the highest-order” network among the *Sociopatterns * datasets. Note that the frequent higher-order interactions observed in the conference setting should not be considered as due to the presentation sessions, which may reunite all participants in one room at the same time. Even if in these situations there are many people in the same room, they are not facing each other. The radio signal of RFID tags are effectively absorbed when crossing the human body (due to the high water content of the body), thus people sitting in rows may not be picked up by RFID tags or identified as face-to-face interactions. Occasions with more higher-order interactions should be instead represented by coffee breaks or poster sessions and therefore they can be considered as actual conversations between the involved users. This may not be true for the proximity datasets recorded by Bluetooth, where large events (like university classes) may induce higher-order interactions. We indeed find much more higher-order events in *CNS* and *F&F* datasets. By pruning contacts on the former we are however able to reduce long-distance co-presence links and make the dataset more similar to the ones only recording face-to-face, see Fig. 2b.

In general, the presence of several group interactions in these networks and their heterogeneous size call for a deeper analysis of their higher-order structures to properly characterize their dynamical evolution.

Moreover, it is interesting to observe that the emergence of higher-order structures is strongly heterogeneous in time. This is evident from Fig. 2c where we show the timely occurrences of interactions of sizes 2, 3, 4 and 5 in the hospital dataset. Note that similar time-series for the other datasets are reported in the Supplementary Information. This visualisation suggests bursty patterns of higher-order interactions, which are not independent across different orders. In one way it is not surprising as higher-order events always build up from lower-order structures, but their heterogeneous dynamics and short term recurrence is far from being obvious. In the following section we will provide a more formal inspection of these features by defining and analysing higher-order bursty behavior.

### Higher-order bursty behavior

To study the dynamics of higher-order interactions we analyze the dynamics of events, which can be a singular interaction or hyperedge of any kind and duration. In the investigated datasets, in order to construct events with longer duration then $t_{res}$, we merge consecutive events which involved exactly the same group of people. In this way we are able to identify longer events with durations modulo $t_{res}$.

We need to introduce an important quantity which measures the time between consecutive events of the same group of people. More precisely, if a generic event *i* begins at time $t_i$ and has duration $d_i$, inter event time $t_{ie}$ is defined as $t_{ie}=t_{i+1}-(t_i+d_i)$. In other words it spans from the end of the group’s previous interaction to the beginning of the next one. Inter-event times are a central measure to study event dynamics as their distribution show whether the dynamics are heterogeneous and thus indicated by a broad $P(t_{ie})$, or they resemble a homogeneous dynamics, such as a Poisson process, with exponential inter-event time distribution^14^. In the Supplementary Information, we report the probability density functions of event durations and inter-event times, respectively in Figs. S4 and S5. These results show that social interactions are strongly heterogeneous in duration and inter-events times regardless the social setting.

To further quantify burstiness in event sequences of different size we measured the burstiness index, defined in Ref.^43^ as $B = [\sigma _{\tau } - \langle \tau \rangle ]/[\sigma _{\tau } + \langle \tau \rangle ]$ where $\langle \tau \rangle$ is the mean inter-event time and $\sigma _{\tau }$ the corresponding standard deviation. This index takes values in (-1,1), with $B=1$ only if $\langle \tau \rangle =0$, representing the most bursty signals where all events are simultaneous; instead $B=-1$ only if $\sigma _{\tau }=0$, i.e. the most homogeneously dispersed signal, the periodic one, with always the same inter-event time. This definition of the burstiness index is however affected by finite size of event sequence, thus it has been corrected for the sample size *n* in Ref.^44^, appearing as an improved version of the original measure: $B_n = [\sqrt{n+1}r - \sqrt{n-1}]/[(\sqrt{n+1} - 2)r + \sqrt{n-1}]$, where $r = \sigma _{\tau }/\langle \tau \rangle$. This measure has no upper bound and, similarly to the previous one, takes the value − 1 for regular signals and identifies temporally correlated events for $B>1$, while $B=0$ in case of independent events. Average values of *B* for the considered datasets and up to interactions of size 4 are reported in Table 1. With the exception of interactions of size 4 in the workplace setting, for which we lack sufficient statistics, all other cases presented appeared with values of burstiness significantly larger than 0. Interestingly, burstiness of events of different sizes appear to be comparable.

**Table 1:** Burstiness measure for distributions $P_s(E)$ reported in Fig. 3.

	Workplace	Hospital	Conference	*CNS*	*F&F*
Size 2	0.58	0.61	0.58	0.31	0.67
Size 3	0.63	0.54	0.62	0.37	0.70
Size 4	− 0.17	0.79	0.70	0.57	0.66

The burstiness has been computed according to the formula firstly proposed by Goh and Barabási^43^ and successively normalised^44^ in order to allow a comparison between samples with different number of events.

The time series reported in Fig. 2 anecdotally suggest that events often occur in successions of high activity, known as *trains of events*, alternated with periods of inactivity. This phenomenon has already been observed for pairwise interactions in various temporal processes^40^, like communication (i.e. emails, text messages or mobile phone calls), recurrent seismic activities in a specific location, and neuron firing signals. It has been argued in Ref.^40^ that the emergence of long bursty trains is ascribable to short-term temporal correlation between events. This can be demonstrated by the distribution of the *E* number of events in the bursty period. To define *E* we need to identify events, which belong to the same bursty period, also called bursty train. In our definition we consider two events to be related if they are consecutive and happen with an inter-event time smaller than a given value $\Delta t$. Related consecutive event pairs can build up to longer trains where the above condition is true for each consecutive event and otherwise the train is separated by longer than $\Delta t$ inter-event times from the rest of the sequence. The number of events in these trains give the metric *E*, whose distribution appears as exponential in case of independent events, while any deviation from this scaling indicates the presence of temporal correlations between the events in the trains. In empirical observations, as mentioned before, the *P*(*E*) distribution has been found to be well approximated by power-law functions, evidently indicating temporal correlations characterising these systems^40^.

**Figure 3:**
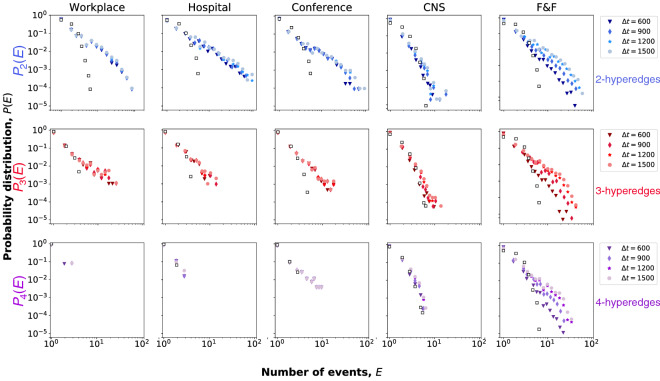
Higher-order structures of temporal trains. Number of events distribution *P*(*E*) for group interactions of fixed size: size 2 (first line), size 3 (second line) and size 4 (third line) interactions. Symbols and colors represent different values of the aggregation window $\Delta t$. Empirical results (full symbols) are compared to the null models obtained by shuffling the event times (empty squares, distributions obtained aggregating with $\Delta t=600$ s).

However, bursty event trains have never been investigated for events involving more than two nodes. Here, we move beyond traditional pairwise interactions and we separate the events according to their size to identify trains of events of each order separately. As earlier defined, we introduce a parameter, $\Delta t$, which allows to discern what we consider related events from uncorrelated ones and to identify event trains. We can identify trains containing only events of a specific size *n* and compute their quantity *E* to obtain the distribution $P_s(E)$ for events of size *s*. Such distributions computed for different event sizes and datasets appear with heavy tails, as shown in Fig. 3. Moreover, this phenomenon appears to be robust against the choice of $\Delta t$ values, coherently with the analysis presented in Ref.^40^. Note that these observations cannot be reproduced by simple null models where event sequences are constructed from uncorrelated interactions obtained by shuffling event times. Equivalent distributions computed in such independent signals are shown on panels of Fig. 3 as empty symbols, appearing evidently different than the empirical observations. For further details on the definition of utilised null models see “Methods” and Ref.^40^.

In summary, our results indicate the existence of bursty dynamics not only for dyadic but also for higher-order event sequences. We observed that they evolve in bursty trains of correlated events in each size of group interaction and in all investigated datasets. More importantly these observations cannot be reproduced by null models of independent events, indicating the observed correlations to be significant in the empirical systems.

### Evolution and formation of higher-order social interactions

**Figure 4:**
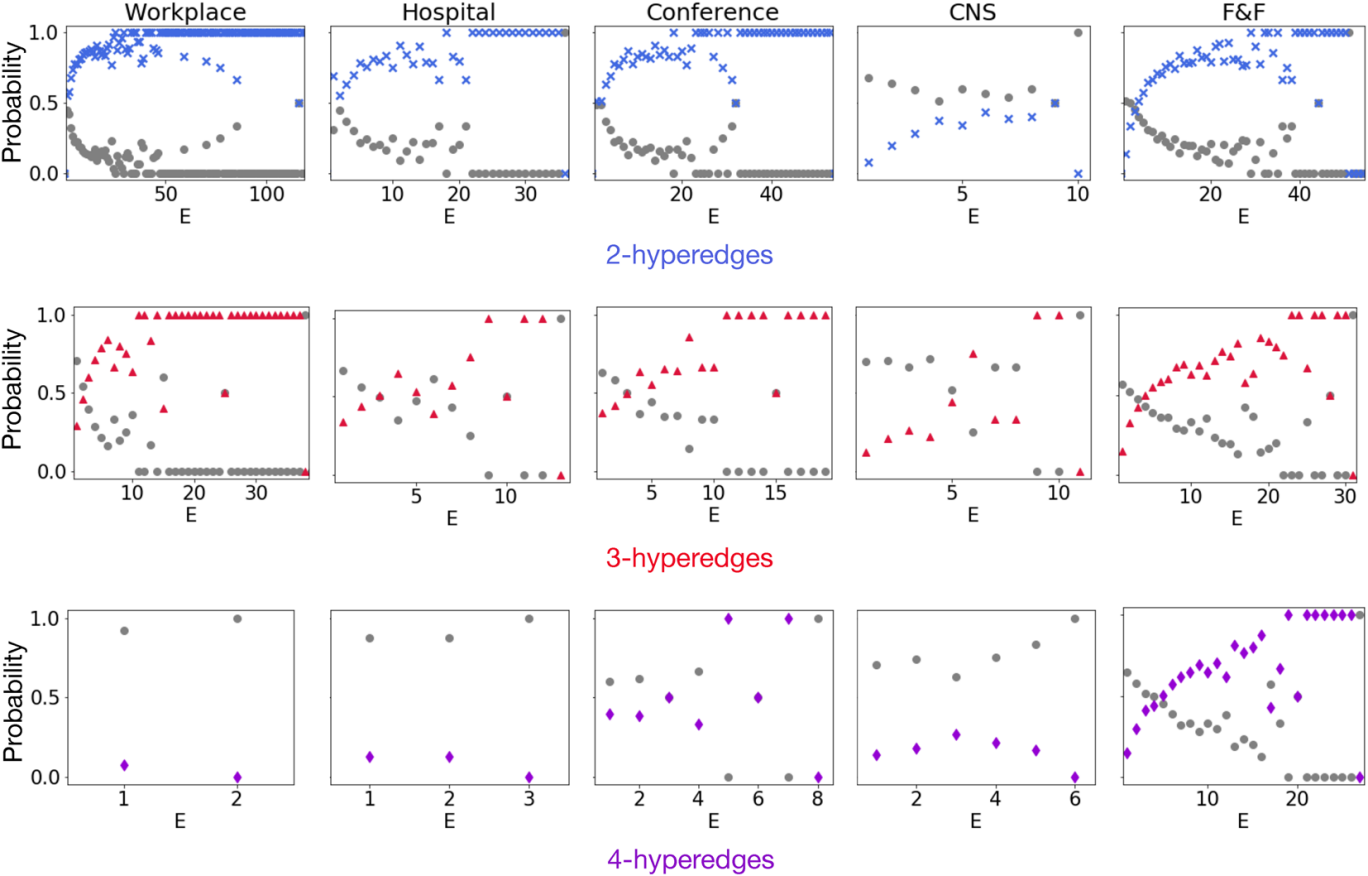
Group evolution and temporal reinforcement of higher-order human interactions. Each panel depicts the probabilities that after a train of interaction at least with *E* events the people involved either interact again in the same or a higher-order structure (coloured symbols) or they do not reconnect anymore (grey symbols) within $\Delta t$. Panels in the first line show results for dyadic trains; panels in the second line for triadic (3-hyperedge) trains; and panels in the third line for 4-body (4-hyperedge). Results are shown for the three analysed datasets for trains identified with $\Delta t = 600$ s.

The above analyses allowed to generalize to higher-order network measures some findings firstly observed in dyadic settings. However, this framework also allows for some genuine higher-order investigation about group formation and evolution, similar to Ref.^45^. The composition of a group in general is related to the previous interaction history of participants, which extends well beyond pairwise relationships. To observe any group formation scenario, we look for the presence of actual interactions in forthcoming time steps thus following how the order of events changes in time. In practice, for events of a given size *s* we consider all the trains of size *E*, and measure the probability that (a) the train continues with an $E+1$th event of the same or higher order or (b) the group falls apart. The results are shown in Fig. 4 extending to higher-order interactions a similar analysis proposed in Ref.^40^ for traditional pairwise communications. Panels in the first line of Fig. 4 depict the evolution of dyadic interactions. For each value of *E* blue crosses indicate the probability that the event is followed by a new event of the same or higher order, while complementary probability, shown as grey dots, measures the probability the corresponding nodes break their interaction in the following time step. Analogous measures are shown in the second and third line for interactions of size 3 and 4, where the two probabilities are shown in color and in grey respectively. Overall, the colored symbols display an increasing trend across the different interaction sizes and datasets. These results indicate the existence of temporal reinforcement, meaning that the longer the length of an interaction—no matter the group size—the higher the chances the relationship will not break down. We note that these trends are more pronounced for groups of small size, which could be due to the significantly larger number of smaller size events in the networks. Although these observations are rooted in some earlier results on group formation dynamics observed through dyadic interactions^11,40,46^, they provide an independent verification of similar phenomena by using higher-order events.

**Figure 5:**
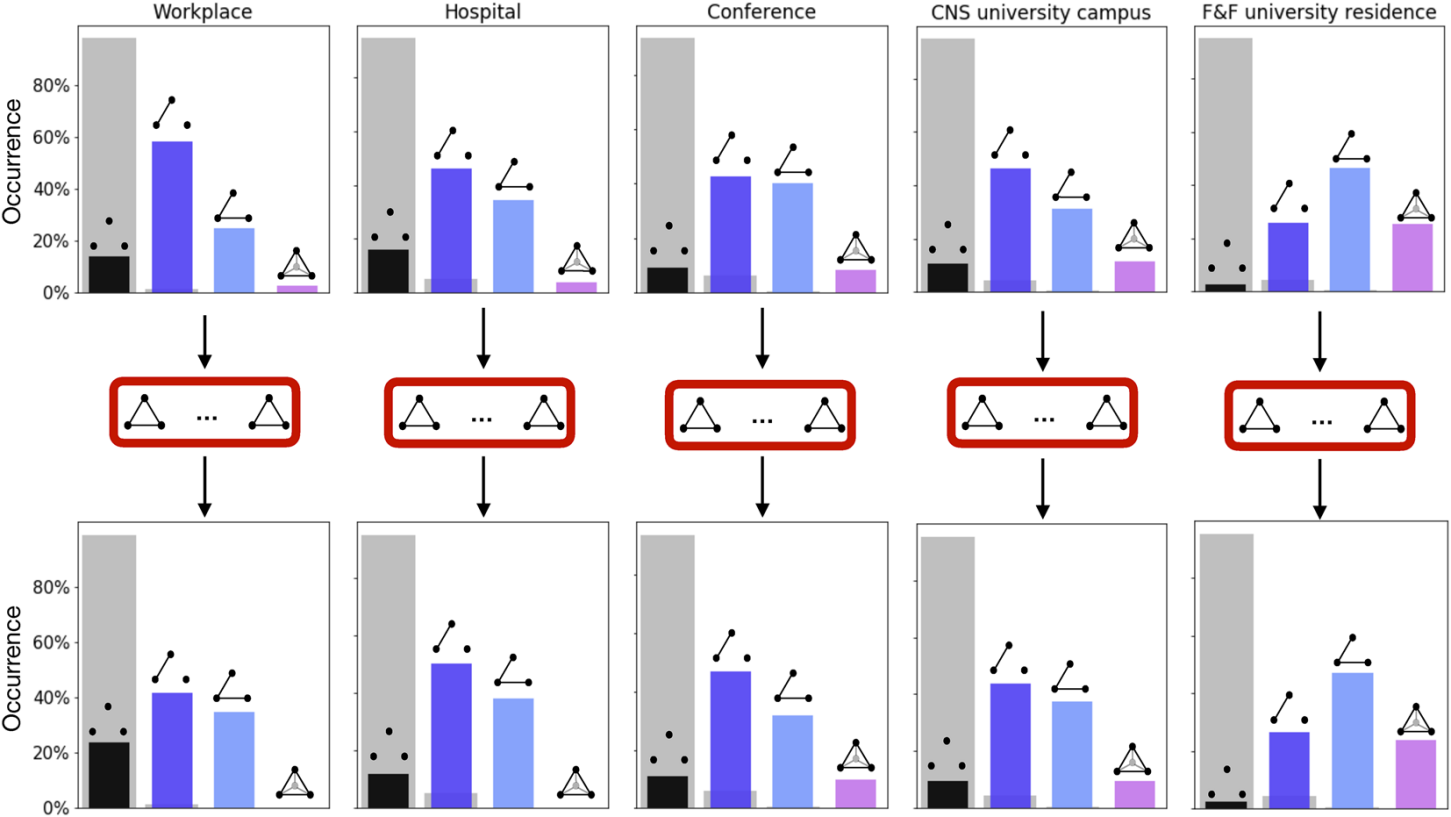
Transition rates of higher-order configurations. Configurations before and after a triplet event lasting at least 4500 s. Four classes of configurations are possible for three nodes that are not part of a 3-hyperedge: all disjoint (black bars), one or two pairwise interactions (dark and light blue bars) or they are all connected and part of larger interaction (purple bars). The grey bars represent the quantity obtained with a null model where temporal graphs snapshots are randomly shuffled.

The question remains, what happens exactly before and after a higher-order event is formed? To answer this question we depart from the exclusive investigation of higher-order events. For each event corresponding to a hyperedge of generic size *n*, we identify the relations among the *n* nodes involved one step before the formation of the group and one step after it disappeared. Out of simplicity, we focus on groups of size 3 (3-hyperedges), as they are by far more numerous within our datasets. At the previous and following time step a clique of three nodes can be arranged across four different classes, as illustrated in Fig. 5 on top of the histogram bars. In the first case there are no connections between any of the nodes (sketch on top of black bars); in the second one there is a single link connecting two of the three nodes, while the last unit is disconnected (sketch on top of dark blue bars); in the third case the nodes are connected across an open triad (light blue bars); and the fourth configuration (purple bars) represents the case where the three nodes are interacting all together but they are part of a larger hyperedge (for this reason they are not classified anymore as a maximal 3-hyperedge). The histograms in Fig. 5 depict the proportions of the four different configurations before (first line of the figure) and after (second line of the figure) a higher-order interaction of size 3 for events lasting at least 4500 s. The grey bars represent a shuffled model where all time correlations are broken by shuffling the snapshots of the temporal graphs (for more details see “Methods”). In this reference model transitions between different motifs are random and the transition probabilities are solely dictated by the overall number of different motifs in the data. In case the originally observed transitions would be only due to the overall motif statistics in the networks, the transition probabilities of the original and randomised systems should closely match each other. However, as demonstrated in Fig. 5, these probabilities are very different, as in the randomized case the most frequent transition is from empty motifs (disconnected nodes), which is not the true for the empirical data.

These results suggest that time correlations frequently induce motifs of one (dark blue) or two (light blue) connected couples before and after a triplet event. Indeed, the corresponding histogram bars are the highest in almost all cases and they are much higher than those obtained with the shuffled model (where the configuration that prevail is the one with three disconnected nodes). In other words the most common configurations are those where two nodes are already connected and a third one is added (dark blue) or, alternatively, two nodes are linked to the same node in an open triad and then they get connected (light blue). Similarly, transition rates for the dis-aggregation of the interaction are high when the triplet is broken in one or two couples. Instead, it appears rather infrequent that a group is created from scratch or vanishes into three isolated nodes (black bars). The two datasets where this is more common are recorded in the workplace and the hospital settings, where we observe a more similar group formation dynamics in the interaction patterns as people may undergo interaction dynamics, dictated by daily work routines. Our finding may be driven by scheduled meetings where a group of people come together suddenly at a given time and then depart. Group formation, instead, appears to be more fluid at the conference, where individuals can connect more freely, and thus it is more common for groups to aggregate and disaggregate step by step, one node at a time. Another important difference between the two kinds of datasets is the formation frequency of larger cliques, indicated by the purple bars. Indeed, in the first two datasets (hospital and workplace) a triplet can in few cases stem from the disintegration of larger groups, but the opposite, i.e. a triplet increasing its size by acquiring new members, never happens. This last possibility is instead common in the conference dataset, where the probability that a group size switches from 3 to 4, or even more, is even higher than the opposite, i.e. a triplet generated from a larger group. This suggests that a triplet is more suitable to represent the starting point of a larger aggregation in the conference setting than in the two working places, and therefore the greater tendency in the former environment to build groups step by step. A similar behavior is observed in *CNS*, which appear closer to the conference setting than to the two working places. The conference and the CNS university campus are indeed two settings where we expect more spontaneous interactions, not entirely governed by scheduled meetings and regular interactions. A different situation is observed in the last column of Fig. 5, depicting *F&F*, the university residential facility dataset with all the co-location contacts that the Bluetooth technology has been able to collect, therefore many more contacts, and not necessarily actual interactions between the involved people. In this case, we observe higher values of the purple bars, coherent with the theory of spontaneity of interactions. Let us notice that the increase of purple bars (and decrease of the black ones) may be influenced by more larger interactions present in these settings. This suggests a tendency to form triplets by starting from pre-existing structures instead of creating them by assembling non-interacting individuals; analogously, triplets tend to evolve into larger or smaller structures, almost never vanishing into three disconnected nodes. This tendency is highlighted again if comparing the colored bars with the grey ones representing the shuffled systems.

In summary, from Fig. 5 we can conclude that in social interactions the temporal evolution towards and from a triplet event is characterized by two common configurations represented by one or two connected couples (respectively dark and light blue). The other two less common possible configurations are the ones with three isolated nodes (black) and that of a larger group (purple), the first one being more frequent in regular settings like those governed by working routines, and the second one observed more in environments characterized by more spontaneous interactions.

## Conclusions

In this work we investigated the dynamics of higher-order interactions in temporal social networks. To this scope, we made use of five publicly available datasets of face-to-face and co-location human interactions collected in different settings as in a hospital, in a workplace, during a conference, in a university campus and in a university residential facility for graduate students and their families. Originally analyzed by means of traditional network tools, the temporal nature of the datasets allowed us to reconstruct the real higher-order organization of social interactions. A first analysis of the datasets revealed the presence of frequent higher-order interactions not limited to simple dyads. More interestingly, such higher-order events appear with heterogeneous bursty dynamics, however with lower frequency for higher-order.

By following the time evolution of the different kinds of interactions we observed bursty trains of higher-order events in all settings. The distributions of bursty train sizes revealed a broad tailed scaling, hinting at similar behavior of higher-order interactions already observed for dyadic events in other bursty systems in biological, geological and social domains^40^. We also inspected memory effects in group formation by measuring the probability that a specific train of interactions is protracted in time, based on the number of previous events and its groups size. We discovered that interactions lasting longer times are more likely to persist even longer, potentially due to temporal reinforcement mechanism characterising all settings.

Group evolution also showed some differences across the considered datasets. In particular for higher-order interactions involving three individuals, we looked at the relational structures at the preceding and forthcoming periods. We found in the hospital and workplace settings similar behavior, possibly due to their work related organisation where individuals are subject to pre-planned and regular dynamics, leading to a higher probability to generate or dis-aggregate groups instantaneously. Differently, in the other settings, we observed a tendency to build groups by progressively adding members, one step at a time, reflecting a more spontaneous way of group formation. A behavior which appears appropriate for a conference, a university campus and a university residential facility, which are suitably characterized by more natural and irregular dynamics.

We can therefore conclude that studying social systems with a higher-order perspective, instead of limit ourselves to observe only pairwise interactions, allows us to seize some fundamental aspects. First of all, the presence of high orders of interactions trivially reveals environments where large aggregations take place. Then, by studying how groups form or disaggregate we can acquire insights into the environments’ dynamics. Indeed, based on the analyzed datasets, we observed that transitions among different group sizes are more abrupt in settings delineated by planned and regular activities and more smooth in environments characterized by more spontaneous contacts.

This study however comes with some limitations. In the Sociopatterns datasets, despite the large amount of captured face-to-face interactions, the investigation of large group behaviors is inherently limited by the lower statistics associated to higher-order events, as compared to pairwise interactions. We tried to overcome this problem by considering additional datasets too, *CNS* and *F&F*, however they have been collected with a different technology and represent co-location instead of face-to-face interactions, with the consequence that some of the collected data could represent casual contacts and not real interactions. Analyzing different datasets increases the richness of the sample but the intrinsic differences between them could be ascribable to their different technologies, making sometimes problematic a comparison.

Overall, our work reveals a new level of richness in temporal human dynamics, neglected in the previous literature. We showed how, taking into account the new framework of higher-order interactions^19^, helps us to better characterise social dynamics extracted from different settings. Taken together we hope that our findings will pave the way to the use of higher-order network tools for investigating the dynamics of human interactions.

## Methods

### Shuffled model

The null model of independent sequences for traditional pairwise interactions is built from the original data by shuffling the times of the events but maintaining all the original pairwise interactions. In this way, we maintain the same time stamps and each node is interacting in the same number of times. Interested readers may look at the comprehensive review on randomized reference models in temporal networks in Ref.^47^, where this model is called as the $P[\mathbf {w},\mathbf {t}]$ timestamp shuffling method.

For group interactions, one possibility is to consider the above defined time shuffled event sequence for each pairwise interaction, and then identify higher-order interactions among them. However this method breaks almost all higher-order patterns and allows the formation of very few cliques of size larger than 2. Hence, we followed another method, where we kept each higher-order event with a given size and shuffled their occurrence times between events of the same size. This shuffling ensures that each event of a given size appears the same number of times as in the original sequence but independently from each other. This is called the $P[p_{\tau }(\mathbf {\Gamma })]$ most random sequence shuffling method in Ref.^47^.

## Supplementary information

Supplementary Information 1

## Data Availability

We are pleased to make available the source-code and datasets accompanying this research. The SocioPatterns data are available at http://www.sociopatterns.org the CNS data at https://doi.org/10.6084/m9.figshare.7267433 and the Friends and family data at http://realitycommons.media.mit.edu/friendsdataset.html.
