# Supplementary material for: Temporal properties of higher-order interactions in social networks
Source: Sci Rep. 2021 Mar 29;11:7028. doi: 10.1038/s41598-021-86469-8 (PMC8007734; doi:10.1038/s41598-021-86469-8)
Supplement: Supplementary file 1 — Supplementary Information 1 [file 41598_2021_86469_MOESM1_ESM.pdf]

Supplementary Information for
Temporal properties of higher-order interactions in social networks

Giulia Cencetti[1] Federico Battiston[2] Bruno Lepri[1] Márton Karsai[2]

[1]Mobs Lab, Fondazione Bruno Kessler, Via Sommarive 18, 38123, Trento, Italy
[2]Department of Network and Data Science, Central European University, 1100, Vienna, Austria

We provide a set of additional figures in order to offer the elements for a more detailed comparison between an approach taking into account higher-order behaviors and a more traditional point of view treating each singular pairwise interaction separately.

Workplace time series

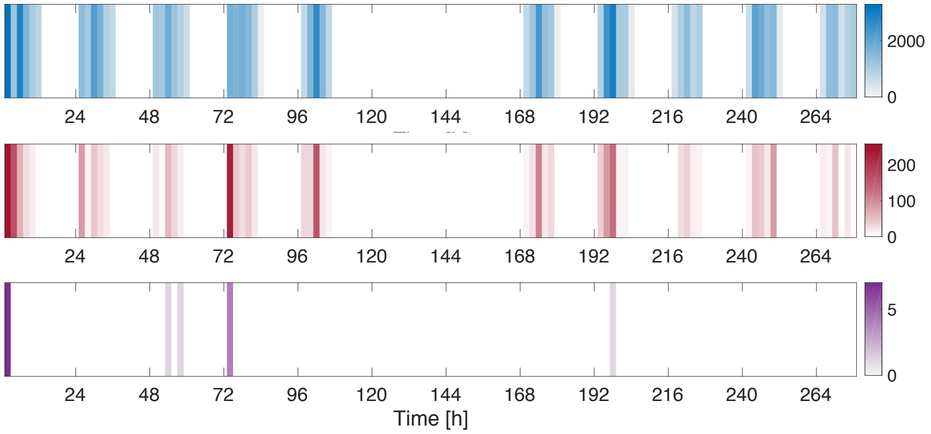

Figure 1: Time series of interactions taking place in an Office building in France [1]. The data have been collected in 2015 for 11 days and the experiment has involved 217 people. The interactions are separated according to their size: blue for 2-hyperedges, red for 3-hyperedges, and purple for 4-hyperedges. We notice a 24h periodicity where couple interactions are the most frequent ones.

Conference time series

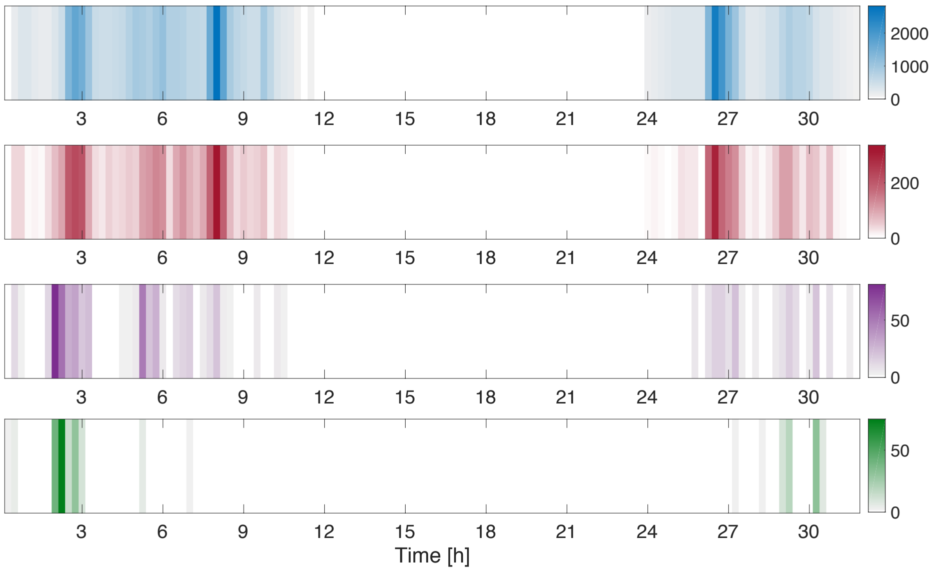

Figure 2: Time series of interactions taking place in the SFHH conference in Nice, France (June 4-5, 2009). The experiment has involved 405 people. The interactions are separated according to their size: blue for 2-hyperedges, red for 3-hyperedges, purple for 4-hyperedges, and green for 5-hyperedges. Larger group interactions are present in this dataset (up to size 9). We notice a 24h periodicity in the two days of the conference and here too we observe that smaller interactions are more frequent.

CNS university campus

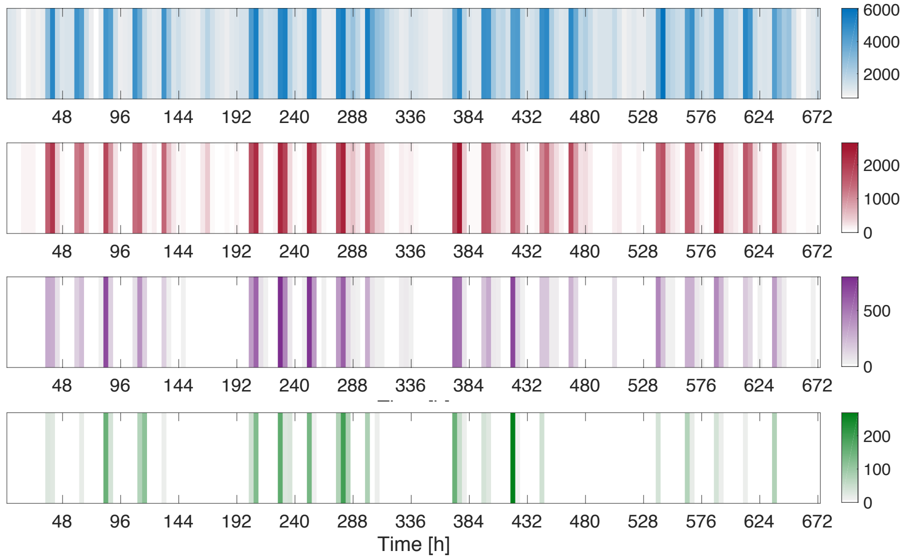

Figure 3: Time series of interactions taking place in the Technical University of Denmark. The experiment has involved 706 students. The temporal network has been pruned in order to only consider close-proximity contacts. The reference period is one month and we can observe a four weeks periodicity. The interactions are separated according to their size: blue for 2-hyperedges, red for 3-hyperedges, purple for 4-hyperedges, and green for 5-hyperedges. Larger group interactions are present in this dataset (up to size 8).

F&F university residence

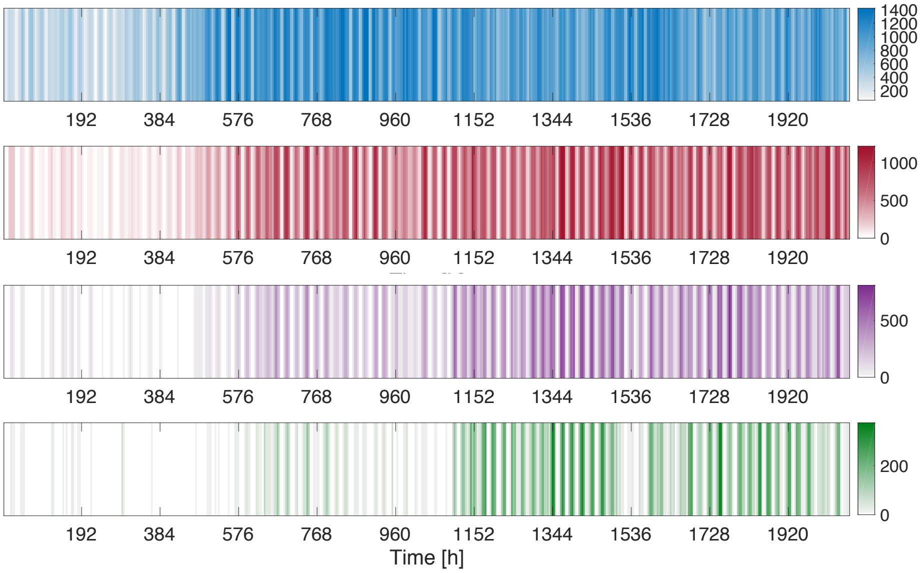

Figure 4: Time series of interactions taking place in a young-family residential living community adjacent to a major research university in North America. All members of the community are couples, and at least one of the members is affiliated with the university, for a total of 130 adults for 15 months. We report here only the first 3 months. The interactions are separated according to their size: blue for 2-hyperedges, red for 3-hyperedges, purple for 4-hyperedges, and green for 5-hyperedges. Larger group interactions are present in this dataset (up to size 14).

Structure of temporal trains of traditional pairwise interactions

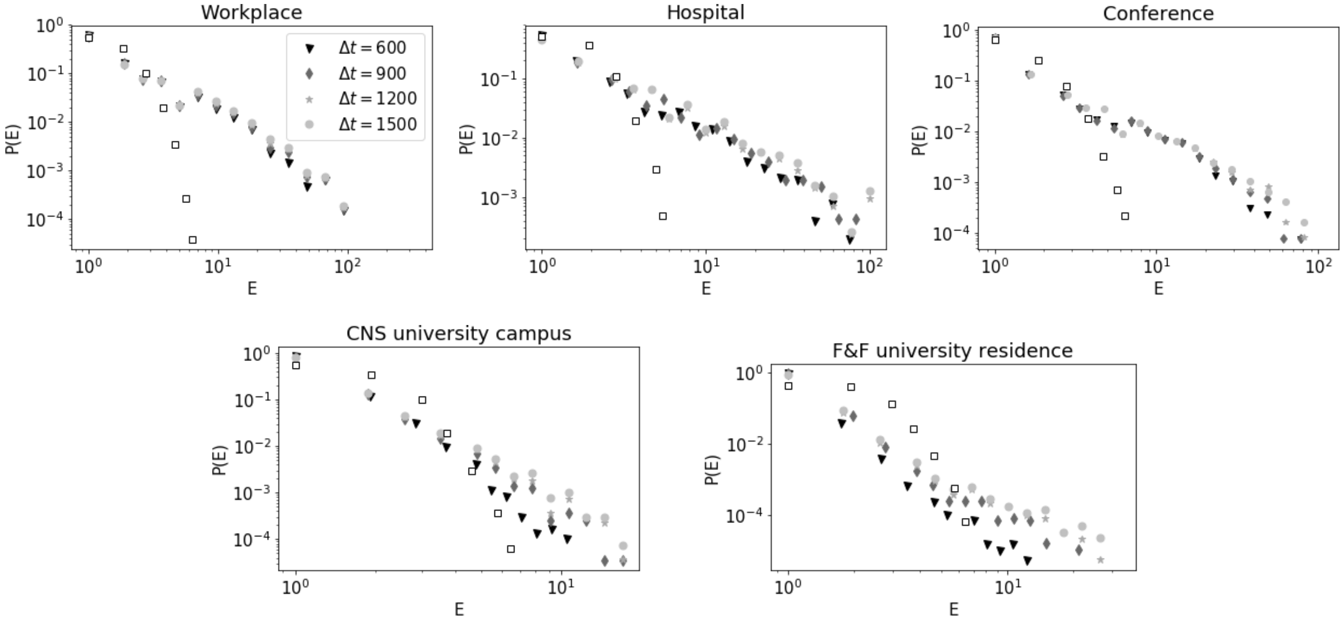

Figure 5: The five panels report the number of events' distribution $P(E)$ in the different social settings, analogously to Fig. 3 in main text, with the difference that here the events are counted considering traditionally pairwise interactions. In details, simultaneous interactions involving a closed group of people are not counted as higher-order interactions but are separated into multiple pairwise interactions. This procedure, which ignores the higher-order nature of social interactions, corresponds to the standard method of analysis related to graphs instead of hypergraphs. For all the three datasets we observe a clear bursty behavior denoted by the power-law shape of the distributions. The distributions are quite independent on choice of the temporal gap Δt and differ from the distribution obtained by the null model (empty symbols), which shows a clear exponential behavior. The null model is obtained with the procedure described in Section Methods of the main text, with $\Delta t = 600$ seconds.

Duration time of events

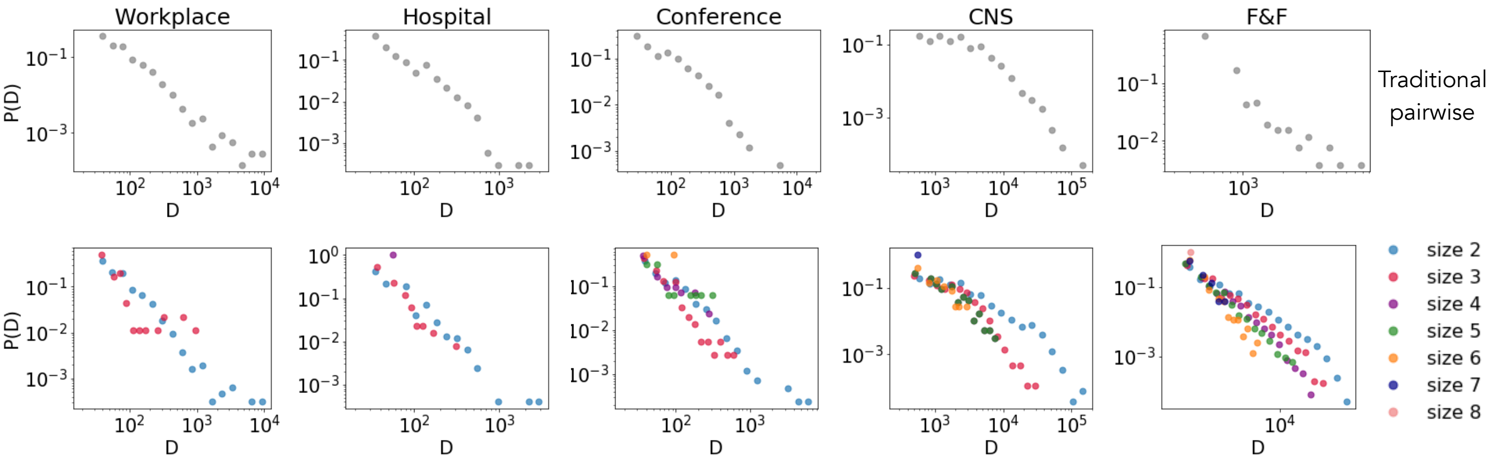

Figure 6: The duration time of events, D, in the traditionally pairwise case corresponds to the length in time spanning from the beginning of an interaction between two people to the end of it. In the higher-order framework instead it represents the time between the appearance of a hyperedge and the disappearance of it, whether it disaggregates in a smaller group or it enlarges its size becoming a hyperedge of a different order. The duration of a higher-order interaction therefore corresponds to the amount of time during which the hyperedge remains unchanged. During the existence of a hyperedge additional links with external nodes can appear but if they do not transform it in a hyperedge of a larger size we consider it unchanged. The figure is organized in panels where the first line of figures shows the distributions of event durations for the five datasets (here the events are counted as traditionally pairwise interactions and not as hyperedges). Analogous figures are also reported in [2]. The second line shows instead how the same distributions appear in a higher-order framework, where interactions are separated according to their size and reported with different colors. We observe that higher-order interactions show duration distributions similar to those found for traditionally pairwise interactions, especially for lower orders.

Inter-event times

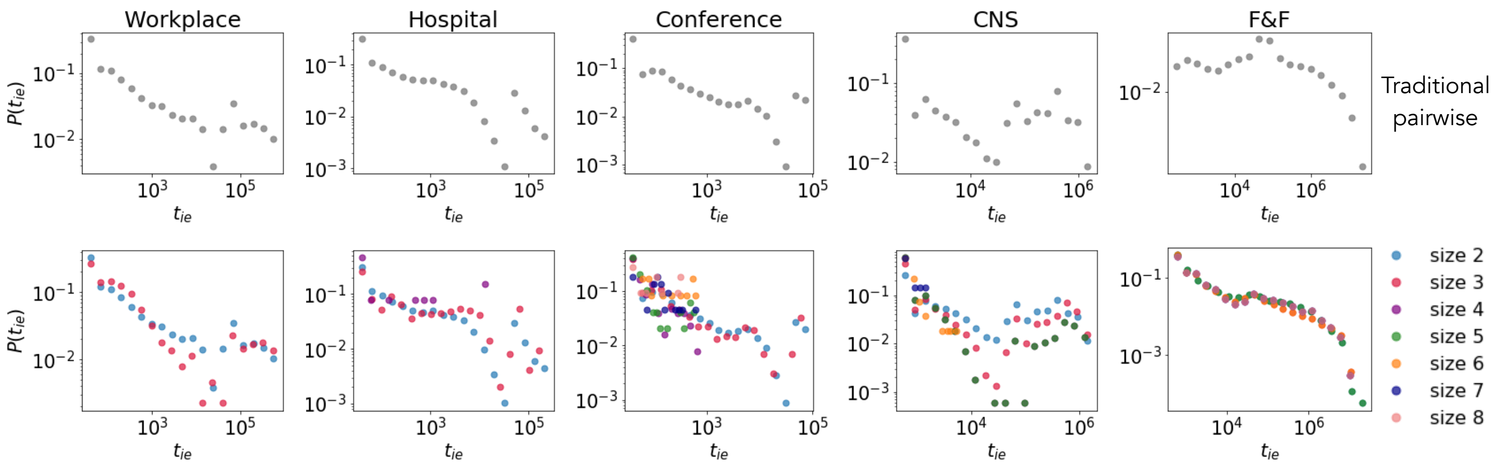

Figure 7: The inter-event time, t_{ie}, is defined as the time elapsing between the end of an event and the beginning of a subsequent event (if existing) involving exactly the same people. Both in the traditional and in the higher-order framework, it corresponds to the time between two different consecutive appearances of the same edge or hyperedge. The figure is organized in panels where the first line shows the distributions of inter-event time for the five datasets, where the events are counted as traditionally pairwise interactions and not as hyperedges. Analogous figures are also shown in [3] and [2]. The second line shows instead how the same distributions appear in a higher-order framework, where interactions are separated according to their size and reported with different colors. We observe that higher-order interactions show distributions similar to those found for traditionally pairwise interactions, especially for lower orders.

Mean size of interactions vs popularity

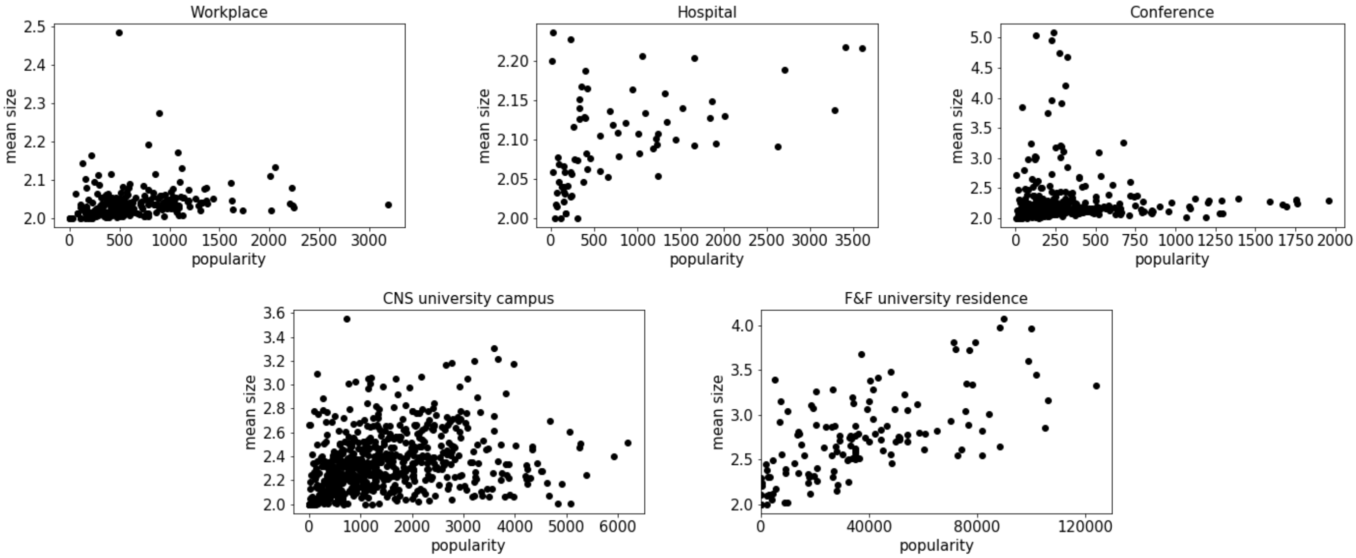

Figure 8: The panels show, for the five datasets, the relation between popularity of each node in the temporal hypergraph and the average size of its relations. The popularity of a node is defined as the total number of interaction events where the node is involved, independently oF their size. Again, we observe similar behaviors in the workplace and the hospital dataset, where the most popular nodes tend to have larger interactions. This time they appear similar to the Bluetooth datasets (CNS and F&F), while the conference setting shows a peculiar result. Indeed, the largest hyperedges are found for nodes with few interaction events: these people tend to have only few but big interactions, maybe people just coming to the conference to give or attending a big talk. Finally, people with a larger amount of interactions tend to participate to small groups, probably people discussing with many others but not participating to big events.

References

[1] M. Génois, C. L. Vestergaard, J. Fournet, A. Panisson, I. Bonmarin, and A. B. Barrat, "Data on face-to-face contacts in an office building suggest a low-cost vaccination strategy based on community linkers," *Network Science*, vol. 3, no. 3, pp. 326–347, 2015.

[2] M. Starnini, B. Lepri, A. Baronchelli, A. Barrat, C. Cattuto, and R. Pastor-Satorras, "Robust modeling of human contact networks across different scales and proximity-sensing techniques," in *International Conference on Social Informatics*, pp. 536–551, Springer, 2017.

[3] M. Karsai, K. Kaski, A.-L. Barabási, and J. Kertész, "Universal features of correlated bursty behaviour," *Scientific reports*, vol. 2, p. 397, 2012.
